# Health phenome of Parkinson’s patients reveals prominent mood-sleep cluster

**DOI:** 10.21203/rs.3.rs-3683455/v1

**Published:** 2023-12-22

**Authors:** Abby Olsen, Joseph Locascio, Idil Tuncali, Nada Laroussi, Elena Abatzis, Polina Kamenskaya, Yuliya Kuras, Tom Yi, Aleks Videnovic, Michael Hayes, Gary Ho, Jordan Paulson, Vikram Khurana, Todd Herrington, Bradley Hyman, Dennis Selkoe, John Growdon, Stephen Gomperts, Trond Riise, Michael Schwarzschild, Albert Hung, Anne Wills, Clemens Scherzer

**Affiliations:** University of Pittsburgh; Center for Advanced Parkinson Research, Harvard Medical School, Brigham & Women’s Hospital; Brigham and Women’s Hospital; Brigham and Women’s Hospital; Brigham and Women’s Hospital; Brigham and Women’s Hospital; Brigham and Women’s Hospital; Brigham and Women’s Hospital; Massachusetts General Hospital; Brigham and Women’s Hospital; Brigham and Women’s Hospital; Brigham and Women’s Hospital; Brigham and Women’s Hospital; Massachusetts General Hospital; Massachusetts General Hospital; Brigham and Women’s Hospital; Massachusetts General Hospital; Massachusetts General Hospital; University of Bergen; Mass. General Hospital, Harvard Medical School; Brigham and Women’s Hospital; Massachusetts General Hospital; Brigham and Women’s Hospital

## Abstract

**Background:**

Associations between phenotypic traits, environmental exposures, and Parkinson’s disease have largely been evaluated one-by-one, piecemeal, and pre-selections. A comprehensive picture of comorbidities, phenotypes, exposures, and polypharmacy characterizing the complexity and heterogeneity of real-world patients presenting to academic movement disorders clinics in the US is missing.

**Objectives:**

To portrait the complexity of features associated with patients with Parkinson’s disease in a study of 933 cases and 291 controls enrolled in the Harvard Biomarkers Study.

**Methods:**

The primary analysis evaluated 64 health features for associations with Parkinson’s using logistic regression adjusting for age and sex. We adjusted for multiple testing using the false discovery rate (FDR) with £ 0.05 indicating statistical significance. Exploratory analyses examined feature correlation clusters and feature combinations.

**Results:**

Depression (OR = 3.11, 95% CI 2.1 to 4.71), anxiety (OR = 3.31, 95% CI 2.01–5.75), sleep apnea (OR 2.58, 95% CI 1.47–4.92), and restless leg syndrome (RLS; OR 4.12, 95% CI 1.81–12.1) were significantly more common in patients with Parkinson’s than in controls adjusting for age and sex with FDR £ 0.05. The prevalence of depression, anxiety, sleep apnea, and RLS were correlated, and these diseases formed part of a larger cluster of mood traits and sleep traits linked to PD. Exposures to pesticides (OR 1.87, 95% CI 1.37–2.6), head trauma (OR 2.33, 95% CI 1.51–3.73), and smoking (OR 0.57, 95% CI 0.43–0.75) were significantly associated with the disease consistent with previous studies. Vitamin supplementation with cholecalciferol (OR 2.18, 95% CI 1.4–3.45) and coenzyme Q10 (OR 2.98, 95% CI 1.89–4.92) was more commonly used by patients than controls. Cumulatively, 43% (398 of 933) of Parkinson’s patients had at least one psychiatric or sleep disorder, compared to 21% (60 of 291) of healthy controls.

**Conclusions:**

43% of Parkinson’s patients seen at Harvard-affiliated teaching hospitals have depression, anxiety, and disordered sleep. This syndromic cluster of mood and sleep traits may be pathophysiologically linked and clinically important.

## Introduction

Parkinson’s disease (PD) is the second most common neurodegenerative disorder. Although a minority of cases are familial, the underlying disease driver for most so-called idiopathic PD cases is unknown. PD is likely to arise through a complex interplay of genetic^1–3^ and environmental factors^4,5^. With a few notable exceptions^6,7^, most epidemiologic studies have largely examined one or a few variables at a time. In the real world, however, patients are much more complex, with phenotypic diversity, varied comorbidities, and polypharmacy. Here we begin to characterize a more holistic picture of clinical, pharmacological, and environmental traits linked to patients with PD.

The Harvard Biomarkers Study (HBS) includes an extensive questionnaire regarding past medical history, medication and supplement use history, social history, and environmental exposures. This includes data on exposure to some previously reported putative risk or protective factors (e.g. smoking, pesticides), though not all (e.g. dairy intake). In this report, we perform age- and sex-adjusted logistic regression to determine which of these health variables are positively or negatively associated with PD. Importantly, these are correlative association and not indicators of causality. Some associations may occur because having the phenotypic variable affects risk of PD, whereas for others the diagnosis of PD affects the risk of the phenotypic variable.

We identify 8 variables (depression, anxiety, restless leg syndrome, obstructive sleep apnea, vitamin D supplementation, coenzyme Q10 supplementation, exposure to pesticides, and history of head trauma) that are positively associated with PD and one variable (smoking) that is negatively associated with PD. Among the variables that are positively associated with PD are several sleep and mood disorders that form part of a larger cluster of correlated variables. Our study represents a rare attempt to begin to characterize the phenome of PD more comprehensively and highlights the high prevalence of sleep and mood disorders in patients with PD.

## Methods

### Harvard Biomarkers Study

The Harvard Biomarkers Study (HBS) is a case-control study including 3,000 patients with various neurodegenerative diseases as well as healthy controls (HC). Informed consent was obtained for all participants. The study protocol was approved by the institutional review board of Mass General Brigham. The HBS questionnaire is included as Supplemental Table 1. For more information on the HBS see: https://www.bwhparkinsoncenter.org/biobank/.

This analysis was limited to a subgroup of the larger HBS, consisting of 1,224 total subjects, 933 with PD and 291 healthy controls based on data availability. Healthy controls consisted chiefly of spouses, friends, and non-blood relatives, who accompanied PD patients to office visits. Diagnosis of PD was made by a board-certified neurologist with fellowship training in movement disorders. Subjects completed a detailed questionnaire including information on past medical history, current and prior medication use, nutritional supplement use, environmental exposures, Parkinson’s disease risk factors, and social history (Supplemental Table 1). Each item in the questionnaire is phrased as a binary yes/no question, which is followed up by quantitative questions in some instances. For example, the question “Do you drink caffeinated coffee?” is followed by questions asking how many cups per day on average and whether the consumption has changed over the past 10 years. For purposes of this initial study, we have limited the analysis to the single binary yes/no question for each variable. Data from the enrollment visit were analyzed. At the time of enrollment, average disease duration was 3.8 years (Table 1).

Statistical differences in demographic data (age, sex, and race) between cases and controls were determined using a Satterthwaite t-test, Chi-square test, or Fisher’s exact test, as appropriate (Table 1). Included in the table are the mean Unified Parkinson’s Disease Rating Scale (UPDRS) and the Mini-Mental State Exam (MMSE) scores. The UPDRS is a 4-part clinical rating scale used to measure severity and progression of PD. In the UPDRS motor subscale (part 3), motor signs were assessed by a trained examiner. A lower UPDRS score indicates better functioning. The MMSE is a 30-question cognitive battery featuring questions on orientation, registration, attention and calculation, recall, language, and copying. A lower MMSE score indicates worse functioning.

### Logistic regression

We tested for association between 64 clinical variables (Table 2, Fig. 4) and PD using age- and sex-adjusted logistic regression analysis in SAS version 9.4. The diagnostic group (PD or healthy control) was the dependent variable. After excluding subjects with missing values, 494 PD and 142 healthy controls were available for this analysis.

### Variable correlation

To determine which subsets of the predictor variables are statistically similar and distinct in this data set, we subjected the Pearson correlation matrix of the entire set of 64 variables to a hierarchical clustering algorithm (employing the Corrplot package in R, version 1.1.423 and the graphical software GraphPad Prism, version 8.43).

## Results

**Figure 1:**
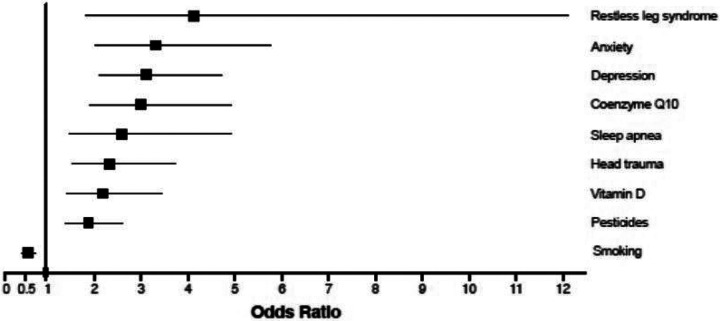
Results of logistic regression. Depression, anxiety, restless leg syndrome, sleep apnea, vitamin D supplementation, coenzyme Q-10 supplementation, head trauma, and exposure to pesticides were over-represented in PD. Smoking was inversely associated with PD. Odds ratio with 95% confidence limit for each variable is shown.

Cases were on average three years older than controls (mean age 66 years for PD versus 63.5 years for healthy controls, with p = 0.0006) and had a higher percentage of males than controls (66.5% vs 43% with p < 0.0001) (Table 1). We thus adjusted our logistic regression analysis for age and sex. 64 features were analyzed for association with PD (Supplemental Table 1). Nine features reached statistically significant associations with PD after adjustment for multiple testing using the false discovery rate (FDR) (Table 2, Fig. 1, Supplemental Fig. 1). Anxiety (OR = 3.31, 95% CI 2.01–5.75), depression (OR = 3.11, 95% CI 2.1 to 4.71), sleep apnea (OR 2.58, 95% CI 1.47–4.92), and restless leg syndrome (OR 4.12, 95% CI 1.81–12.1) were significantly more common in patients with Parkinson’s than in controls adjusting for age and sex with FDR ≤ 0.05. Exposures to pesticides (OR 1.87, 95% CI 1.37–2.6), head trauma (OR 2.33, 95% CI 1.51–3.73), and smoking (OR 0.57, 95% CI 0.43–0.75) were significantly associated with the disease consistent with previous studies. Vitamin supplementation with cholecalciferol (OR 2.18, 95% CI 1.4–3.45) and coenzyme Q10 (OR 2.98, 95% CI 1.89–4.92) was more commonly used by patients than controls.

Five additional variables had *suggestive* associations with PD with nominal *P* values below 0.05 adjusting for age and sex (Supplemental Table 2) but did not meet our statistical significance threshold correcting for multiple testing. Three of these variables had suggestive positive associations: the NSAID ibuprofen (OR 1.74, 95% CI 1.13–2.77), bupropion (OR 10.28, 95% CI 2.18–183.84), and antiemetic use (OR 3.09, 95% CI 1.17–10.67). Two variables had suggestive inverse associations: ezetimibe (OR 0.13, 95% CI 0.023–0.73) and multivitamin supplementation (OR 0.71, 95% CI 0.54–0.94).

**Figure 2:**
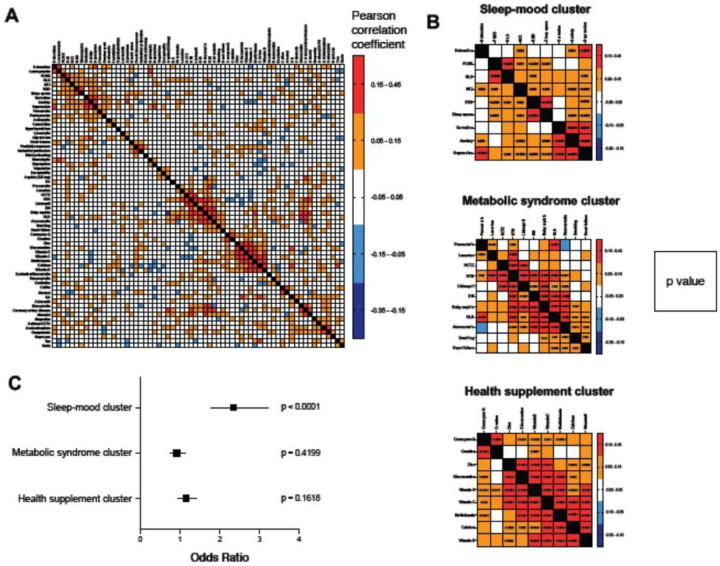
Heat map demonstrating correlations between the individual clinical and environmental variables. A. Pearson correlation matrix. B. Several sets of variables are positively correlated, forming clusters of biologically related phenomena. The largest clusters in our dataset include a linked sleep and psychiatric factor cluster, a metabolic syndrome cluster, and a vitamin use cluster. P-values indicating the significance of the correlation are shown in the boxes. C. Of the 3 clusters, only the sleep-mood cluster is significantly associated with PD.

To exam whether some of these features are correlated and thus may tag the same underlying trait we clustered the pair-wise correlation structure between the 64 variables. The resulting Pearson correlation matrix is shown in Fig. 2A. Hierarchical clustering of the correlation coefficients revealed three correlated feature clusters (Fig. 2B).

*Cluster 1* represents nine correlated *mood and sleep traits*. The mood disorders are depression and anxiety. The cluster members sertraline, a serotonin reuptake inhibitor, and duloxetine, a serotonin and norepinephrine reuptake inhibitor are medications for treating both anxiety and depression. The sleep disorders in the cluster are REM sleep behavior disorder (RBD), which is a well-known non-motor manifestation of PD that may be linked to dysfunction of the locus coeruleus^8^, which accounts for nearly all norepinephrinergic projections to the substantia nigra, basal ganglia, and cortex^9^. It also includes periodic limb movements of sleep (PLMS) and sleep apnea, which are common in PD patients^10,11^. Interestingly, restless legs syndrome (RLS) is also a member of this cluster. RLS responds to dopamine replacement medications, and patients with RLS often have accompanying PLMS^12^. Within this cluster, depression was the variable with the greatest degree of correlation (based on correlation coefficients and p-values) to all other variables in the cluster.

*Cluster 2* represents *metabolic syndrome traits*. It includes the cardiovascular and metabolic diseases of hypertension, hyperlipidemia, diabetes mellitus, and heart failure. Additionally included are two cholesterol lowering medicals (pravastatin, atorvastatin), 3 anti-hypertensive medications (losartan, lisinopril, and hydrochlorothiazide) and baby aspirin. Interestingly, smoking, which is frequently presumed to be tightly linked to COPD, was a prominent member of this metabolic syndrome cluster without correlation to COPD (rho = 0.02). Within this cluster, hypertension was the variable with the greatest degree of correlation to all other variables.

*Cluster 3* represents nine correlated *health supplements*. The high degree of correlation between various vitamin supplements is consistent with our clinical impression that patients who take vitamins are likely to take multiple vitamins.

To determine if the 3 clusters of variables were themselves associated with PD, we repeated the sex and age adjusted logistic regression after combining the variables within each cluster into a single factor score. A factor score for each person was defined as the proportion of the variables within that factor that a person possessed. Excitingly, the mood-sleep factor score (cluster 1) was significantly associated with PD (Fig. 2C), while the other two clusters were not appreciably associated with PD. This is consistent with the view that sleep and psychiatric features are phenotypically and possibly etiologically linked in PD.

**Figure 3:**
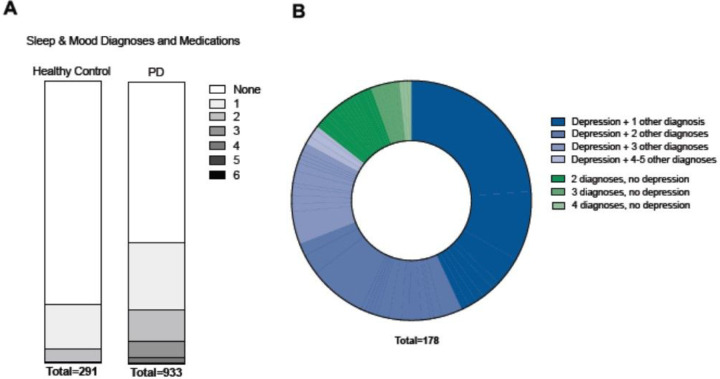
High prevalence of sleep and mood disorders in PD patients. A. PD patients are more likely than healthy controls to have multiple sleep and mood disorders. B. Among PD patients with multiple sleep and mood depression, a large majority have depression.

Cumulatively, 42% (396 of 933) of Parkinson’s patients had at least one psychiatric or sleep disorder, compared to 21% (61 of 291) of healthy controls. Among the variables contained in the sleep-mood cluster, depression was the single most prevalent diagnosis in PD patients, with 24.5% of PD patients (229 of 933) having a diagnosis of depression, compared to 11.7% of controls (34 of 291). Further, 20% of PD patients (187 of 933) had multiple sleep-mood cluster diagnoses, whereas only 5.5% of healthy controls (16 of 290) had multiple diagnoses (Fig. 3A). Among the PD patients who carried more than one diagnosis in the sleep-mood cluster, a large majority of 81% (152 of 187) had depression as one of the diagnoses (Fig. 3B, Supplemental Table 3).

## Discussion

**Figure 4:**
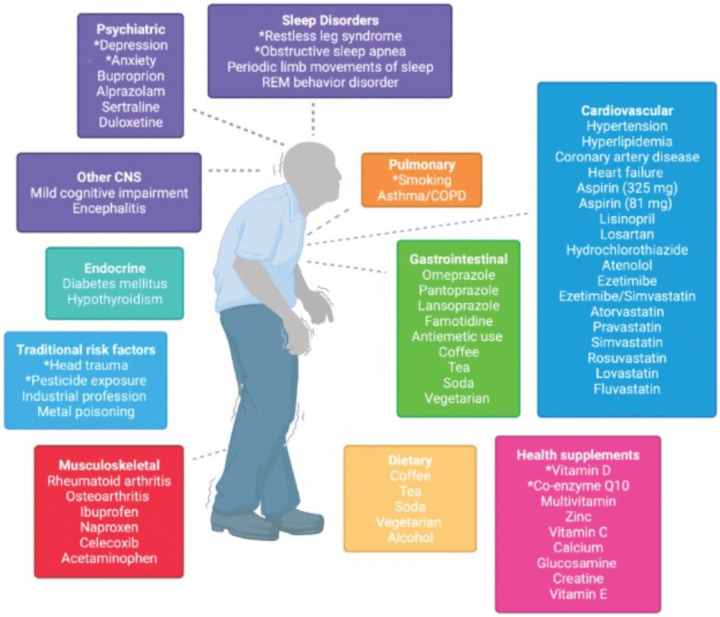
Schematic demonstrating the variables examined in this study. Variables span domains of past medical history, medication and supplement use, social history, and environmental exposures. They capture functions of many organ systems. Variables that were significant in the logistic regression are indicated with *.

The goal of this study was to comprehensively characterize the complexity and heterogeneity of clinical issues faced by people with PD seen in movement disorders clinics in North America. This study represents a multi-dimensional and data-rich view of clinical associations with PD (Fig. 4). As this is a case-control study, the results should be interpreted as associations — not causality. That is, while some associations may arise because a phenotypic variable affects risk of PD, others almost certainly arise because of having a diagnosis of PD affects risk of the phenotypic variable.

One striking finding in our analysis was the strong prevalence of a history of mood and sleep disorders in our PD population. The interplay between mood disorders and PD is complex, with some evidence suggesting that these disorders represent either pre-motor^13^ or very early non-motor^14^ co-comorbidities. Similarly, sleep disorders are prevalent in PD^15^ and may arise either prior to^16^ or after the onset of motor symptoms^17^. REM behavior disorder (RBD) in particular may represent a prodromal state of PD, and there is growing interest in conducting clinical trials in patients with isolated RBD given the high rate of phenoconversion^18^.

Mood disorders, sleep disorders, and PD may share some common pathogenic mechanisms, including degeneration or dysfunction of dopaminergic, serotonergic, and noradrenergic circuits^19–21^. PD is defined by loss of dopaminergic neurons in striatonigral pathway, with relative preservation of the mesocortical and mesolimbic pathways. Patients with major depressive disorder also have dopaminergic disturbances, which may improve with treatment with anti-depressants^22^. Further, the dopaminergic system plays a role in circadian rhythm, initiation of REM sleep^23^, and RLS, which is treated effectively by dopamine agonists. Beyond dopamine, serotonergic neurons of the raphe nuclei^24^ and noradrenergic neurons in the locus ceruleus^25^ also degenerate in PD. Serotonergic dysfunction is the primary pathogenic driver of major depressive disorder and may also be linked to sleep to sleep disorders^26^. Noradrenergic signaling is dysregulated in both depression and anxiety^27^, and it controls sleep-wake states^28^. Thus, mood disorders, sleep disorders, and PD all feature combinations of dopaminergic, serotonergic, and noradrenergic dysfunction.

One emerging idea is that PD patients with sleep and mood disorders may represent a distinct clinical phenotype of PD, that is the “brain-first” as opposed to “body-first” subtype^29^. Others have posited that there is a noradrenergic subtype of PD, noting the high coincidence of REM behavior disorder, pain, anxiety, and dysautonomia in a subset of patients^30^. These observations highlight the importance of screening PD patients for mood and sleep disorders, which have a substantial impact on quality of life and are amenable to treatment^31^.

Our study confirms an inverse association between smoking and PD. Of all environmental factors that have been inversely associated with PD, the relationship between smoking and PD is perhaps the best established. This was documented as early as the late 1960’s^32^ and has been reproduced by numerous subsequent epidemiologic studies^33^. The mechanisms underlying this association are unknown, though there is ample evidence for a protective role of nicotine in dopaminergic neurons in animal models of PD^34–37^. In clinical trials of PD patients, treatment with nicotine, the active ingredient of cigarettes, however, failed to achieve disease modification^38–40^. Interestingly, in our data, smoking was part of the cardiovascular cluster 3 (Fig. 3B), but not correlated with asthma/COPD (R = 0.02, p = 0.7163). Smoking was significantly correlated with baby aspirin use (R = 0.08, p = 0.004), atorvastatin use (R = 0.11, p = 0.007), hyperlipidemia (R = 0.08, p = 0.0001), and heart failure (R = 0.08, p = 0.005). Thus, the common assumption that COPD/asthma is a generally useful marker for smoking is not reflected in our data set. Cardiovascular disease has been associated with development with PD^41^ and with increased risk of progression^42^, but studies examining the relationship of statin use to pd^6,43–46^ have yielded mix results.

No statin was significantly associated with PD in our dataset, though the non-statin cholesterol-lowering drug ezetimibe had a nominally significant p-value suggesting an inverse association with PD (Supplemental Table 2). These results highlight the need for epidemiologic studies to consider complex interactions and links between cardiovascular traits, cardiovascular medications, and smoking on each other and on PD.

Our study also supports previously reported associations between head trauma and pesticide exposure and PD. The evidence for a positive association between head trauma and PD has been mixed. While several studies including a 2013 meta-analysis have demonstrated an association^47–49^, multiple large population level Scandinavian studies have not^50–52^. Recall bias and the timing of injury are potential complicating factors^53–54^. Thus, further studies are needed. Similarly, while several large epidemiologic studies have linked pesticide exposure to PD risk, including the Honolulu Asia Aging Study, the Cancer Prevention Study-IIN, and the Agricultural Health Study, in most studies exposure is self-reported, and the role of individual pesticides is unclear.^5^ The strongest evidence for pesticides comes from the mitochondrial complex I inhibitors, rotenone and paraquat^55^.

There are also limitations of our analysis. For example, we previously detected an inverse relationship between quantified caffeine intake and PD^56^ in HBS. Caffeine intake was carefully quantified^56^ in the prior analysis. Participants reported their usual consumption of caffeinated and decaffeinated coffee, tea, and soft drinks during the previous 12 months in standard volumes, with 9 possible frequencies ranging from never to 6 or more cups (for coffee and tea) or cans (for soft drinks) per day. Mean daily caffeine consumption was calculated based on standard food composition sources. In contrast, here we considered only a binary yes/no for each of the exposures considering the large number of variables analyzed. Further, although the HBS questionnaire is extensive, it is not exhaustive. Some medications such as asthma inhalers were not recorded in HBS until recently. Thus, we could not evaluate for associations between asthma/COPD^57,58^ or asthma medications^59–61^ and PD, which have been identified in several recent studies. This highlights the need for future studies examining variables quantitatively rather than categorically.

We have chosen to focus our discussion on the variables that were statistically significant after adjusting for sex and age and after correcting for multiple tests. Beyond these, we found some variables that were nominally statistically significant but did not survive correction for multiple testing (Supplemental Table 2). These results should be interpreted with caution and will require confirmation in other patient cohorts. Discordant findings may be due in part to suppression effects, in which positively or negatively correlated variables (Fig. 2) may suppress or overestimate each other’s true relation to PD.

In summary, here we have provided an initial comprehensive clinical characterization of PD patients in the HBS. Our results confirm some previously reported associations as well as highlight other novel associations. Many of the health variables we have examined here are modifiable, meaning that these results may someday have implications for personalized medicine^62^. Future work will require mechanistic studies to identify gene-environment interactions, to determine which factors are truly causative, and to discover whether modifying them has a neuroprotective or symptomatic benefit. As one of the few patient cohorts with this extensive collection of environmental exposure data combined with whole genome sequencing, the Harvard Biomarkers Study represents a transformative resource for holistically decoding the environmental, genetic, biological and clinical features of PD.

## Figures and Tables

**Table 1:** Clinical characteristics. Statistical differences in demographic data (age, sex, and race) between the cases and controls were determined using Satterthwaite t-test, Chi-square test, and Fisher’s exact test, as appropriate. UPDRS, Unified Parkinson’s Disease Rating Scale. MMSE, Mini-Mental State Exam.

Characteristic	Healthy controls	Parkinson’s disease	p-value
Sex			<0.0001
Male	125 (43%)	620 (66.5%)	
Female	166 (57%)	312 (33.5%)	
Age at enrollment (years)	63.5 ±11.7	66.1 ±9.9	0.0006
Race			
Asian	2 (0.7%)	9 (0.7%)	1.000
Black/African American	13 (4.9%)	9 (0.7%)	<0.0001
Hawaiian/Pacific	1 (0.3%)	0	0.19
More than one race	3 (1 %)	3 (0.2%)	0.09
Unknown	4 (1.4%)	38 (3.1%)	0.12
European	268 (92.1%)	1166 (95.2%)	0.04
Ethnicity			
Hispanic	8 (2.7%)	17 (1.4%)	0.12
Non-hispanic	277 (95.2%)	1168 (95.3%)	0.88
Unknown	6 (2.1%)	40 (3.3%)	0.34
Education (years, S.D.)	15.0 ±1.77	15.2 ±1.76	0.16
Age at onset		62.3 ±10.4	
Disease duration		3.8 ±4.4	
Hoehn and Yahr stage		2.1 ±0.64	
UPDRS motor subscale		19.8 ± 10.2	
UPDRS total		33.2 ±15.1	
MMSE	28.99 ±1.26	28.21 ±2.30	<0.0001

**Table 2:** **Features associated with PD.** P-value, odds ratio, 95% confidence limit, and prevalence in HC versus PD are shown. Each variable is controlled for sex and age.

Feature	*P*-value (age/sex adjusted)	FDR	Odds ratio	95% C.I.	Healthy controls	Parkinson’s disease
Restless leg syndrome	0.0027	0.019	4.12	1.81–12.1	5 (3.0%)	76 (12.6%)
Anxiety	<0.0001	0.0013	3.31	2.01–5.75	18 (6.5%)	140 (15.8%)
Depression	<0.0001	0.0013	3.11	2.1–4.71	34 (12.2%)	229 (25.7%)
Coenzyme Q10	<0.0001	0.0013	2.98	1.89–4.92	22 (7.6%)	170 (18.2%)
Sleep apnea	0.0019	0.015	2.58	1.47–4.92	13 (4.5%)	115 (12.3%)
Head trauma	0.0002	0.0021	2.33	1.51–3.73	25 (9.1%)	176 (19.8%)
Vitamin D	0.0007	0.0064	2.18	1.4–3.45	34 (23.6%)	202 (40.3%)
Pesticides	0.0001	0.0013	1.87	1.37–2.6	63 (22.5%)	316 (35.7%)
Smoking	<0.0001	0.0013	0.57	0.43–0.75	143 (50.9%)	368 (41.5%)

## Abbreviations

PD: Parkinson’s disease
HBS: Harvard Biomarkers Study
